# Integrated Care for Frail Elderly: A Qualitative Study of a Promising Approach in The Netherlands

**DOI:** 10.5334/ijic.4626

**Published:** 2019-09-03

**Authors:** Maaike Hoedemakers, Fenna Ruby Marie Leijten, Willemijn Looman, Thomas Czypionka, Markus Kraus, Hanneke Donkers, Esther van den Hende-Wijnands, Nicole M.A. van den Broek, Maureen Rutten-van Mölken

**Affiliations:** 1 Erasmus School of Health Policy and Management, Erasmus University Rotterdam, NL; 2 Institute for Advanced Studies, Vienna, AT; 3 Department of care and innovation, Care group DOH, NL; 4 Care group PoZoB, NL; 5 SGE, Eindhoven, NL; 6 Institute for Medical Technology Assessment, Erasmus University Rotterdam, NL

**Keywords:** integrated care, frail elderly, multi-morbidity, patient-centeredness, continuity, SELFIE

## Abstract

**Introduction:**

Increasingly, frail elderly need to live at home for longer, relying on support from informal caregivers and community-based health- and social care professionals. To align care and avoid fragmentation, integrated care programmes are arising. A promising example of such a programme is the Care Chain Frail Elderly (CCFE) in the Netherlands, which supports elderly with case and care complexity living at home with the best possible health and quality of life. The goal of the current study was to gain a deeper understanding of this programme and how it was successfully put into practice in order to contribute to the evidence-base surrounding complex integrated care programmes for persons with multi-morbidity.

**Methods:**

Document analyses and semi-structured interviews with stakeholders were used to create a ‘thick description’ that provides insights into the programme.

**Results:**

Through case finding, the CCFE-programme targets the frailest primary care population. The person-centred care approach is reflected by the presence of frail elderly at multidisciplinary team meetings. The innovative way of financing by bundling payments of multiple providers is one of the main facilitators for the success of this programme. Other critical success factors are the holistic assessment of unmet health and social care needs, strong leadership by the care groups, close collaboration with the healthcare insurer, a shared ICT-system and continuous improvements.

**Conclusion:**

The CCFE is an exemplary initiative to integrate care for the frailest elderly living at home. Its innovative components and critical success factors are likely to be transferable to other settings when providers can take on similar roles and work closely with payers who provide integrated funding.

## Introduction

Like many countries with a rapidly ageing population, the long-term care sector in the Netherlands is transitioning to improve efficiency and maintain affordability [1,2]. During the major reform in 2015 the long-term care sector was decentralised. The number of nursing homes was reduced considerably and access to nursing homes was restricted to those in need of 24-hour care. Municipalities became responsible for the provision of domestic home care and social support, whereas healthcare insurers became responsible for nursing care at home [3]. The reform stimulates elderly who were previously eligible for residential care and admission to nursing homes to stay at home longer, signalling the increased focus on self-sufficiency in our society. At the same time, the home care sector faced significant budget cuts [3].

As a result of the reform, a greater proportion of (frail) elderly is living at home with the support of primary care, home care, and informal care [3,4]. This population increasingly has a combination of physical, social and mental health problems [5]. Although, ageing in their own homes is generally in line with the preferences of elderly people, it also creates challenges [5,6,7,8]. The greater involvement of the municipalities in the funding of domestic and social care requires good communication and coordination between health and other care providers in order to prevent fragmentation or duplication that may lead to inefficient and ineffective care [9,10]. The collaboration between these providers is hampered by the traditional segmentation and ‘silo-thinking’ that is embedded in all aspects of the system [11,12]. There is no single professional or organisation that is truly responsible for coordinating care and support. Although GPs often take on this role, they do not always have a sufficient overview of, and time to explore, all available support services [13]. The increased complexity of frail elderly still living at home increases the number of visits to emergency departments and unplanned hospital admission and complicates the transfer of care when people return home [13,14]. Furthermore, the reform increased reliance on informal caregivers and thus alongside the possible benefits (e.g., feeling good about supporting a loved one, giving more meaning to one’s life) the burden on these individuals increases (e.g., health problems, social isolation, withdrawal from the workforce) [14,15,16,17,18]. These challenges highlight the importance of improving the coordination of care within and across sectors in order to ensure efficient and high quality care.

With the long-term care reform in sight, care providers in the Netherlands developed integrated care programmes for frail elderly. The development of these care programmes was stimulated by the Ministry of Health, which commissioned the National Care for the Elderly Programme that provided project-grants, and by healthcare insurers who offered additional funding for innovations [19,20,21,22,23,24,25,26,27,28]. The rise of these care programmes is accompanied by the need to evaluate such programmes, as healthcare insurers require evidence on their effectiveness in order to move from temporary to structural funding. However, these programmes are complex interventions and difficult to evaluate: they consist of multiple interacting components, target multiple levels (individuals, groups, organisations, and systems), have a variety of intended outcomes that are largely impacted by the behaviour of those delivering and receiving the interventions, and are continuously adapted and improved [29,30,31]. Moreover, they often involve some form of budget pooling to break down the silos within and between health and social care. Previous studies have shown that elements from integrated care programmes are not always appropriately or fully implemented, or they worked out differently when put into practice [32,33,34].

The current paper provides an analysis of a thick description of a promising integrated care programme, the Care Chain Frail Elderly (CCFE), which is being implemented in the Netherlands. The CCFE is one of 17 innovative integrated care programmes being investigated in the EU-funded Horizon2020 SELFIE project (see **Box 1**). SELFIE aims to stimulate evidence-based implementation of integrated care for persons with multi-morbidity. The CCFE was selected because most frail elderly have multi-morbidity (i.e., co-occurrence of two or more chronic health conditions within one individual). The CCFE particularly met our selection criteria of being innovative in actively involving the individuals with multi-morbidity, their informal caregivers and the social care sector [41], which is what many integrated care initiatives are striving for. Sharing our in-depth understanding of the CCFE acquired through qualitative research may help to achieve this.

The purpose of this paper is to highlight the innovative elements of the CCFE and the factors that contribute to its success. It also aims to create awareness of the challenges involved in the implementation of the CCFE and how to address them. This leads to important insights that may inform future efforts to develop similar programmes in different settings and design evaluation studies.

**Box 1: Information on the SELFIE project**

**SELFIE** (Sustainable intEgrated chronic care modeLs for multi-morbidity: delivery, FInancing, and performancE) is a Horizon2020 funded EU project that aims to contribute to the improvement of person-centred care for persons with multi-morbidity by proposing evidence-based, economically sustainable, integrated care programmes that stimulate cooperation across health and social care and are supported by appropriate financing and payment schemes. More specifically, SELFIE aims to:

- Develop a taxonomy of promising integrated care programmes for persons with multi-morbidity;
- Provide evidence-based advice on matching financing/payment schemes with adequate incentives to implement integrated care;
- Provide empirical evidence of the impact of promising integrated care on a wide range of outcomes using Multi-Criteria Decision Analysis;
- Develop implementation and change strategies tailored to different care settings and contexts in Europe, especially Central and Eastern Europe.

Seventeen promising integrated care programmes for persons with multi-morbidity are being evaluated in SELFIE using MCDA and a common set of core outcomes as well as programme-type specific outcomes. The latter depend on whether a programme is i) a population health management programme, ii) a programme targeting frail elderly, iii) a programme targeting persons with problems in multiple life domains, or iv) an oncology or palliative care programme.

The SELFIE consortium includes eight organisations in the following countries: the Netherlands (coordinator) (NL), Austria (AT), Croatia (HR), Germany (DE), Hungary (HU), Norway (NO), Spain (ES), and the UK. (www.selfie2020.eu) [Grant Agreement No 634288].

## Methods

### Study design

In this study we qualitatively described a single case study applying a thick description: a qualitative empirical research method to investigate implicit social practices, such as care delivery, in their specific contexts [35]. A thick description covers several depths of analyses. The starting point is a formal description of the ‘hard facts’ based on document analyses. These written documents are often not sufficient to give a deeper understanding of what actually constitutes the programme below its surface when put into practice, i.e. the ‘soft facts’ on the ‘how’ and ‘why’. For this purpose semi-structured interviews with key stakeholders are conducted. The interviews also complement the hard facts gathered in the course of the document analyses. When writing this manuscript we adhered to the COnsolidated criteria for REporting Qualitative research (COREQ) [36].

### Procedure and data collection

The thick description method used in this study was centrally developed by the Austrian partner in the SELFIE consortium. During a SELFIE-meeting, they trained each partner-country in conducting thick descriptions. Specifically, interviewers were trained in using interview protocols and analysing the results. The one-day training focused on identifying relevant stakeholders, compiling interview protocols, and different methods of qualitative content analysis.

We studied a variety of documents about the care programme: official documents and contractual documents related to the programme, documents related to past evaluations, presentations given by project leaders, factsheets about the care programme and the collaboration between the care groups, a business case, documents regarding the bundled payment and other financial agreements, and documents about specific working groups related to the care programme. Most documents were provided by the project leader of the CCFE, others were publicly accessible on the internet.

For the interviews, we invited a purposive sample of 13 stakeholders via e-mail and/or phone. Two persons refused due to time constraints. Hence, over a 3-month period (July–September 2016), 11 semi-structured, face-to-face interviews were conducted with initiators of the care programme (n = 2), programme managers (n = 3), representatives of the payer organisations (n = 2), medical and social care staff (n = 2), an informal caregiver and a patient. An overview of the stakeholders and their reference is given in Appendix A.1. Interviews with professionals took place at their workplace, with the informal caregiver and patient they took place at their home. Interviews took between 33 and 62 minutes (mean 49 minutes). Five interviews were conducted by the first author (MH) and six by the first author together with a co-author (FL). No other persons were present during the interviews besides the interviewee and interviewer(s). These interviewers had a minimum of a Master’s degree and experience in patient-contact and qualitative research. Prior to the interviews, authors had no established relationships with the interviewees; only with the programme managers there had been prior contact in order to prepare the participation of the CCFE in SELFIE and to identify stakeholders to interview.

For the different types of stakeholder groups, thematic focus areas were pre-defined across all SELFIE thick descriptions (see Appendix A.2), and a set of protocols for semi-structured interviews was prepared by the Austrian team and adapted to country/programme specific issues by the Dutch team of SELFIE. By interviewing different types of stakeholders, we could gain insights into the programme from various perspectives. Interviewees were sent a topic list prior to the interview. Before the start of the interview, the interviewer(s) briefly introduced themselves and the SELFIE project. When new themes arose during the interviews, these were used in the interviews to come. The interviews were audio-recorded and transcribed verbatim. The transcripts were not returned to participants for correction, but the interviewees were sent the thick description and the quotes we used in the thick description.

### Data analysis

All information retrieved from the document analysis was structured according to the conceptual framework for integrated care for multi-morbidity that was developed at the beginning of the SELFIE project, see Appendix A.3 [37]. In the core of the framework is the holistic understanding of the person with multi-morbidity. This is surrounded by six components to systematically describe a care programme: service delivery, leadership & governance, workforce, financing, technologies & medical products, and information & research. The first author analysed the transcripts and discussed findings with two co-authors (FL and MRvM). Analysis was done using Mayring’s content analysis method [38]. The transcripts were coded using mostly deductive coding as the topics were largely determined a priori. For each of the components of the framework, and for each topic described within the framework, sentences and paragraphs were selected that supplemented or illustrated the existing text. When new topics came up during the interviews, these were also coded and transformed into constructs. The first and second author separately coded the transcripts. The first draft of the thick description report was sent to the Austrian partner (TC, MK) and to the last author to provide feedback on the findings.

The thick description of the CCFE can be found on the SELFIE website. The analysis presented in this manuscript focuses on the most innovative elements of the CCFE that characterise the programme.

### Ethics statement

The Medical Ethical Committee of the Erasmus Medical Centre Rotterdam declared that this research was exempt from the Medical Research Involving Human Subjects Act. Participation was voluntary and could be retracted at any point. All participants signed an informed consent form, which was developed on the basis of the WHO informed consent for qualitative research and consisted of the following information: brief description of the SELFIE project, purpose and type of the research, participant selection, voluntary participation, procedure, duration, potential risks, benefits, reimbursements, confidentiality, sharing of the results, right to refuse/withdraw, and contact information.

## Results

The CCFE targets community-dwelling frail elderly with complex care needs.

Frailty is defined as a loss of functional abilities and control over one’s life due to case and care complexity, which requires multidisciplinary care and case management. Subsequently, case complexity is defined as having complicated diseases, disabilities and frailty – often occurring simultaneously and difficult to diagnose. Care complexity refers to complicated care, for example due to a combination of needed care and no informal caregiver being present.

**Figure 1:**
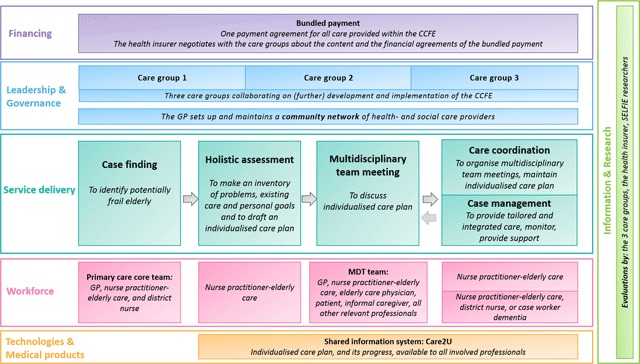
Care process Care Chain Frail Elderly. *Note: MDT = Multidisciplinary team*.

An overview of the programme and its components can be found in Figure 1. The CCFE programme started as a pilot in 2011 in a selected group of general practitioners. From 2013 onwards a wider implementation took place. The general goal of the frail elderly care programme is to provide person-centred care coordination and case management to keep frail elderly at home for as long as possible. An additional aim, formulated from the payers’ perspective, is to develop structured multidisciplinary primary care that decreases the demand for secondary care, postpones nursing home admissions, and reduces health care costs for persons in this stage of life.

Below, the most noteworthy aspects of the CCFE are described per component of the conceptual framework.

### Service delivery

The CCFE is organised into four phases: (1) proactive case finding, (2) holistic assessment, (3) multidisciplinary team meeting(s), and (4) care coordination/case management. Three key aspects are discussed below.

#### Case finding

Potentially frail elderly are identified by a primary care core team of professionals using a case finding approach with inclusion criteria as defined above in a non-quantitative way. This approach includes a home visit to get a good overview of the health and social care needs. There is an agreement with the health care insurer that only the top 1% frail elderly of a GP-practice will be included in the programme. The insurer does not require a specific diagnosis for reimbursement, and instead trusts the professionals to select the elderly that will benefit most from the care programme.

An advantage of this approach is that professionals are granted a lot of responsibility for including the ‘right’ patients. The central role of the primary care core team stimulates multidisciplinary collaboration between the GP-practice and home care. However, the ‘soft’ and un-protocoled method of selecting frail elderly can lead to issues; variations in the inclusion criteria across professionals have been observed, which is at the expense of uniformity. This variation partly results from the geriatric tool that is used to perform a holistic assessment of somatic, psychological, social, and communicative life-domains, as well as general functioning and self-sufficiency. Some professionals use this tool as a secondary inclusion criterion, whereas other professionals use the tool merely for guidance. One professional gave an example of an older person scoring high on the tool, but not being frail because the patient was handling the situation well. This professional argued that scoring is one thing, but the conversation between the professional and elderly is another:

“The instrument provides an indication of a situation, not necessarily an indication of problems. We should avoid being paternalistic in our approach.” (PM_1)

Thus, although the current case finding approach is likely to identify patients most in need of support, it may also increase practice variation.

#### Multidisciplinary team meetings

During the multidisciplinary team (MDT) meetings the individual care plan is discussed so that all professionals are aware of the elderly’s goals, and the types of care/support the frail elderly person receives. This aims to ensure that all care providers are working towards the same goal in a proactive manner. Examples of interventions include: consultations with e.g., physiotherapist or dietician, weekly visits to a day care centre for elderly, arranging that a volunteer can help with e.g., groceries, medication review(s) by a pharmacist, or informal caregiver support.

The MDT-meetings distinguish the CCFE from similar care programmes, because the frail older person and his/her informal caregiver are also invited to participate. Initiators, professionals and the patient and informal caregiver stress the importance of the involvement of the patient in the MDT-meeting for several reasons (*MS_2, IC_1, PM_1, MS_1, and FE_1*). The frail older person mentioned that he appreciates it and sees that all the professionals are collaborating with one-another. If anything was incorrect, he could “*jump in, and everyone listened to you*.” (*FE_1*) The informal caregiver stated that as a result of the multidisciplinary meeting:

“people at least communicated with one-another and looked at what was […] really necessary for us”. (IC_1)

They further felt that they could be involved in the goal setting process. The patient and informal caregiver, and several other stakeholders, highlight the importance of their presence at the meeting to ensure a high quality of care and patient-centeredness and -involvement.

Downsides of their involvement, however, are that for patients it could be overwhelming to attend a meeting with all their caregivers at once, that the professionals need to adjust their professional language to the level of the patient (which could make the meeting take longer), that scheduling issues arise when multiple busy professionals and the patient and informal caregiver need to be present, and that MDT-meetings can only be used to discuss one case at a time. Furthermore, the healthcare insurer argue that if the patient is in good contact with his/her case manager, this should be sufficient. One of the initiators, who is also a care provider, stated that the added value of the patient’s presence at the MDT-meeting remains inconclusive:

“Whenever the patient wishes to participate in the multidisciplinary team meeting, it will always be useful. However, it is not necessary for the patient to hear about technical details.” (IN_2)

Although more evidence on the added benefit of the patient’s involvement in the MDT may be needed, it is hard to isolate the effect of this single component of the CCFE.

#### Care coordination and case management

The care programme separates two main care tasks. Care coordination supports the patient and his/her informal caregiver in keeping an overview, and navigating through the system. As most of the frail elderly’s care is complex, multiple professionals are involved, which enhances the care burden for that patient. Case management monitors the execution of the individual care plan, signals additional needs and further adapts the care to the patient’s wishes and needs.

Both tasks are usually carried out by the same professional, the nurse practitioner specialised in elderly care, which is beneficial for the continuity of care. This is not a requirement and there is a degree of flexibility. In some cases, the district nurse is appointed as case manager, usually because she was already involved in the care process and is the caregiver who visits the elderly most frequently. An advantage of having this professional take on this role, is that collaboration between the GP-practice and home care organisations, where the district nurse is employed, is stimulated. Despite this, initial evaluations revealed difficulties in the collaboration between the district nurse and the GP-practice. One of the difficulties relates to data-sharing. District nurses need to record data in in the shared information system ‘Care2U’, as well as in their own information system, causing duplicate registration. Another difficulty is that GPs have to work with several district nurses in their community, and the turnover of district nurses is rather high. Thus, GPs have to maintain many collaborations and give many district nurses access to their agendas. As time passed, close physical proximity to the GPs and the electronic medical records appeared to result in fewer district nurses fulfilling the tasks of case manager.

### Leadership and governance

Key elements of leadership and governance are the role of care groups and the community networks (see Figure 1).

#### Care groups

Unique to the CCFE, and perhaps more generally to Dutch primary care provision, is the role of care groups. A care group is a group of primary care providers that cooperate in the provision of chronic care and support GPs in implementing care pathways. Healthcare insurers contract a care group, and not the individual GPs. Care groups either employ or subcontract professionals who provide the care.

Three care groups in the Netherlands developed the CCFE. This governance has given the care groups a strong position to negotiate with the healthcare insurer about the content, price and quality of care. Alongside the benefits in relation to cross-disciplinary collaboration and financing, the collaboration between the three care groups also enhances the uniformity of frail elderly care within the region, because they aligned their ideas surrounding frail elderly care. Using a uniform approach is important, especially since many professionals are involved:

“A district nurse has to deal with several GP-practices and the GP-practices have to deal with several home care organisations and different teams. To make it even more complicated, there are just a lot of parties. That is why we decided to work in a uniform way.” (IN_1)

Although uniformity is achieved to a large extent, differences between the care groups and between the types of GP-practices remain. One example is the difference in organisational culture and leadership. The fact that care groups either employ or subcontract the professionals results in different scopes of influence a care group has on the care being provided. Furthermore, some GPs are running a practice in a small village, whereas others work in a large group practice in a big city, affecting the efforts required to create community networks.

#### Community network

As the target population consists of frail elderly living at home, a great amount of cross-sector collaboration from formal and informal care providers is required. So far, professionals in the network had primarily been working alongside one-another without actually collaborating. The reforms of the long-term care sector in the Netherlands tried to stimulate collaboration between health and social care. The CCFE has embraced this trend and required that GPs set-up community networks. Professionals recognise the importance of a close collaboration between sectors. One of the district nurses describes the benefits:

“The good thing about our collaboration is that we know each other very well. When I’m with a client and I notice something special, I can call the GP and I know that I will be heard, because we know each-other.” (MS_1)

The ease and effectiveness of collaboration between GP-practices and the social care sector, specifically with district nurses, differs between GP-practices. A professional responsible for setting up a community network mentions that it may be easier to work in a small town, in a smaller setting, because it is easy to identify possible partners in the care-chain to collaborate with (MS_2). Also, for some GP-practices, the collaboration with home care organisations was already established before the start of the CCFE, making it easier to reach out to these organisations (MS_1). As a result of implementing the CCFE, this collaboration became more structural with meetings being held on a regular basis (MS_1). However, the collaboration with the welfare organisations, which for example provide community-based volunteer-support, is not yet optimal for reasons related to privacy protection when sharing information and the large amount of these organisations. Nevertheless, the importance of establishing community networks is widely recognised.

### Workforce

In the CCFE a differentiation is made between the primary care core team and a wider network of professionals that can be called upon in the multi-disciplinary care team.

#### New multidisciplinary teams of professionals

The *primary care core team* consists of the GP, nurse practitioner and district nurse, and meets once or twice a month to discuss potentially frail elderly. The role of this team is to signal frailty within- and outside of the healthcare sector and match care accordingly.

The *multidisciplinary care team* discusses the individualised care plan based on the personal goals of the elderly. Possible professionals involved are the nurse practitioner, GP, district nurse, elderly care physician, physical therapist, psychologist, case worker dementia, pharmacist, speech therapist, occupational therapist, and/or geriatrician. Whomever is already involved in the care process, is invited to attend the MDT-meetings, including the older person and his/her informal caregiver. This ensures person-centred, integrated and coordinated care as all care providers agree on the same care plan.

#### Key professionals

The nurse practitioner is one of the key professionals in the core team. This nurse has followed an educational programme to specialise in elderly care. In the CCFE she is involved in each step of the care process, works in close collaboration with the GP, and also maintains contact with other professionals involved in the care process. For most frail elderly she is the main contact point. Tasks appointed to the nurse practitioner in the CCFE are: case management, care coordination, setting up a community network (in collaboration with the GP), and process monitoring of transfer care, polypharmacy, and data collection of quality indicators on the patient level. She has a certain amount of hours to spend on elderly care, next to her other tasks as nurse practitioner.

The elderly care physician plays an important advisory role in the multi-disciplinary care team. This is a relatively new medical professional working in primary care in the Netherlands. (S)he is specialised in frail elderly care, and has experience working in multidisciplinary teams and with advance care planning. The elderly care physician has an important role in the programme in coaching the GP and nurse practitioner and acting as a source of information for them. (S)he reviews the results of the holistic assessment and the individualised care plan and is available for home visits and consultations with other professionals. The rationale behind involving these professionals in the CCFE is that they will improve the knowledge and skills of the GP and nurse practitioner, which will gradually make their own role smaller over time.

The structural embedding of both care teams into the CCFE and the important role of the key professionals has greatly stimulated cross-sector collaboration and they are seen as valuable assets.

#### Role of the informal caregiver

At the national level there is a trend towards a greater role for the informal caregiver; the CCFE aims to unburden the informal caregiver by recognising the potential burden and ensuring adequate support to prevent drop-out of the informal caregiver and hospitalisation of the frail elderly. One of the goals of support is to ensure that the positive aspects of informal caregiving (satisfaction) outweigh the burden. According to one of the professionals, elderly are not always aware of the severity of their frailty and the amount of support they need from their informal caregiver(s) (MS_2). The role of the nurse practitioner is then to convince the frail elderly to accept formal care:

“We are trying to meet the needs of the patient, yet also to unburden the informal caregiver and increase the safety of the patient.” (MS_2)

### Financing

To incentivise integration of care, the programme is funded via a bundled payment contract that each care group negotiates separately with the healthcare insurer. The bundled payment is a fixed annual budget that should cover all frail elderly care. It is based on three factors: an average tariff per frail elderly based on the estimated number of minutes of care, agreed upon between insurer and care group (care group specific, confidential), overhead costs (care group specific, confidential), and an estimated number of frail elderly included in the programme (care group specific). It is agreed upfront that this number should not exceed 1% of the GP-practice. The bundled payment covers care provided by the GP, nurse practitioner, pharmacist (for medication review), geriatrician (consultation by phone), and the physician assistant. It also includes tasks not directly related to a patient, e.g., setting up the community network. It does not include care provided by the district nurse, elderly care physician, case manager dementia, physiotherapist, occupational therapist, social worker and welfare worker. These professionals are funded in the usual way, either by the healthcare insurers (Health Insurance Act) or by the municipality (Social Support Act).

An important facilitator to the implementation of the CCFE were the macro-level reforms that supported this exploration of new ways of financing elderly care. The bundled payment is a great improvement in financing, both compared to the fee-for-service payment for consultations in the past and to the short-term project based way of financing integrated elderly care. It is seen as an innovative and sustainable way of financing integrated care for community-dwelling frail elderly. The advantage of the bundled payment from the perspective of an insurer is that it allows them to contract the care group, and care programme, as a whole, instead of contracting all individual GPs and care activities separately (HI_2). Not only does this result in a lower administration burden, the insurer can also delegate the monitoring of the care delivered by the GP to the care group. The insurer believes it is easier for a care group to steer and monitor a GP, since a care group is managed by care providers that easily relate to the GPs (HI_2). Furthermore, the bundled payment results in predictable costs for the insurer because the size of the target population is predictable and the costs of the bundled care are known. However, the bundled payment contract has to be renewed annually and the burdensome negotiations about what care to include and for which tariffs start over again. Nevertheless, the key elements of the care programme have not changed much since the beginning:

“In 2010/11 we invented the care programme, and at this moment, the key elements are the same. […] We still think it is best to include the patient in the multi disciplinary team meeting, and the goals in the individualised care plan are the patient’s goals and the professional’s… These elements remain always a topic of discussion [with the insurers], but we keep coming back to the same quality requirements.” (PM_1)

Both the care groups and the insurer do have plans about the further development of the CCFE. The insurer encourages the care groups to further differentiate the reimbursement for GPs, based on the case mix of patients in their practice. The care groups do not see a fundamental reason to do so, as it goes against the basic idea of integrating payments and it would increase the difficulties in administration (*PM_3*). The care groups would rather see that all activities not directly related to the care programme – for example treatment of ear syringing – are reimbursed outside the bundled payment. The healthcare insurer does not want to reimburse all consultations separately, because it is hard to define the boundaries of frail elderly care.

The influence of the insurer has been a challenge, but is also an asset as it provides promise for the financial sustainability of the programme. A debated point between the insurer and care groups is the continuous request of convincing effectiveness evidence. Furthermore, the presence of the frail elderly and informal caregivers at the MDT-meetings is being debated (*HI_1, HI_2*) (see also *Service delivery*). In contrast to the professionals who are generally very positive towards their presence, the healthcare insurer has pointed out the lack of evidence on the benefits, making it difficult to secure funding for this element of the programme (*HI_2*). After speaking with frail elderly and informal caregivers in other regions where they are not involved in the MDT-meetings, the insurer did not get the impression that these elderly were any less satisfied and these programmes were less costly (*HI_1*).

Finally, it has to be acknowledged, that although the bundled payment incentivises collaboration between professionals, the scope of the current bundle is limited. It only includes reimbursement for a few professionals, mostly operating in primary care. In the future it would be desirable to expand this.

### Information and research

The healthcare insurer has emphasised that evaluation plays an indispensable role in the future of the bundled payment and discussions with the care groups (HI_1). Very preliminary findings from a qualitative study by the insurer suggested that there is room for improvement in the way the ICT is organised and the collaboration with social care. Overall, the direction of the results were quite positive, which was beneficial for the continuation of the care programme.

The care groups have also tried to evaluate the programme. They can extract quality indicators from the shared information system, but also collected data from a small sample of patients. However, these evaluations had limitations concerning the number of respondents, lack of control group, and the small scope of outcomes. Although all parties are aware of the importance of evaluating the care programme, they also recognise the difficulties, e.g., selection bias, measuring patient-reported outcomes (PROMs) and experience (PREMs) in frail elderly, and spill-over effects making it difficult to identify an appropriate control group.

### Technologies and medical products

In the CCFE the information system ‘Care2U’ is used, that offers a secured platform to share information between the professionals involved. There is a direct link between the GP information systems and Care2U, which saves a lot of time. Professionals not working at the GP-practice, however, have to log their proceedings in their own information system separately. Also, a district nurse working with GPs from the three different care groups, has three accounts to log into Care2U. These difficulties are seen as challenges:

“It is possible for the various chain partners to use Care2U, but to make it work on an organisational level, there should be financial incentives. For example, that registration for reimbursement is done in Care2U.” (PM_1)

Each individual care plan is posted in Care2U and is accessible for all involved professionals. The frail older person needs to approve that professionals can access Care2U. Professionals have different degrees of access; some professionals only need to have access to specific information, such as a dietician, whereas other professionals need to have access to all information. Care2U enables the nurse practitioner to monitor appointments and tasks of partners in the care chain, for example to see if these professionals have met certain deadlines (e.g., for lab results).

The frail elderly can make use of a patient portal that was developed to support self-management. It is not yet possible to link this patient portal to Care2U, and thus does not provide access to the individualised care plan. One of the care groups has started a pilot to create this link and offers elderly the opportunity to report their experience in the individual care plan directly (PM_1).

Having a shared information system for professionals and a patient portal are two potentially influential facilitators for the CCFE to succeed. Nevertheless, if these systems create extra work, they may function as a barrier. Thus, ensuring that professionals can easily use these and that they save time will make the programme attractive and efficient.

## Discussion

In this study we systematically described and analysed a promising integrated care approach for frail elderly along the lines of the six components of the SELFIE framework for integrated care for persons with multi-morbidity. The CCFE programme has several factors in common with other integrated care programmes for frail elderly, e.g., a holistic assessment, individualised care plan, multidisciplinary care, care coordination, and/or case management [19,20,21,22,23,24,25,26,27,28], but is innovative and distinguishes itself in that it targets the *frailest* GP-population, invites the frail elderly and their informal caregivers to participate in the MDT-meeting, and is funded through a new bundled payment system.

### Challenges and facilitators

One of the challenges of the care programme during implementation was the harmonisation of the case finding process across care groups in order to ensure inclusion of a similar population of frail elderly. Relying on professional judgement may be an efficient way to ensure inclusion of the frail elderly most in need of better support and most responsive to change. In the current approach, elderly are included when they are already very frail. In the future it might be desirable to identify elderly with an increased risk of becoming frail earlier on, in order to focus more on prevention and create better long-term results [39].

Part of the case complexity of the frailest elderly is related to their social environment. Therefore, the development of a community network requires further attention. The care programme offers time and funding for this task. District nurses seem to be well equipped for this because they have a good overview of services offered and collaborating with other parties in the community is part of their everyday work [40]. However, GPs still struggle with initiating and maintaining collaborations with the social care providers because they do not have much experience with this.

The presence of the patient and informal caregiver at the MDT-meeting is the ultimate expression of person-centred care and crucial for shared decision-making. Although it is an essential element of the care programme, it complicates planning of the meetings and increases their duration and costs, and could therefore be a barrier to sustainable implementation.

The CCFE has the means to address these challenges. Namely, a dedicated staff and management, the bundled payment and the Care2U ICT-system. Extending the bundled payment to include a wider variety of services is possible, especially for healthcare services covered by the healthcare insurers. The inclusion of social care services is more difficult as it would require breaking down the funding silos between health and social care, the latter of which is covered by the municipality. The Care2U ICT-system facilitates cross-sector collaboration because all professionals involved in the care provision have access to it, with different disciplines having access at different levels. Compatibility with ICT-systems outside the care groups is a hurdle to overcome. Its further development is funded by the bundled payment.

### Strengths and limitations of the study

This in-depth qualitative analysis of the CCFE programme, incorporated the perspectives from multiple stakeholders, including professionals, managers, payers, a patient and an informal caregiver. This contributed to a broad insight into the evolution of the programme in daily practice, which commonly deviates from the plans on paper. We used a purposeful sample of interviewees and we did not continue recruiting respondents until data saturation was reached. However, we did get a broad overview of different views although it is possible that more critical views are less well represented. Nevertheless, it is not the aim in qualitative research to attain a representative sample.

Furthermore, our analysis encapsulates a moment in time in the continuous adaptation and improvement of the programme. Examples of adaptations currently being implemented are optimisation of transfer care to and from hospitals, more frequent medication reviews, better integration of dementia care, and further development of a patient portal to give the elderly access to their individual care plan. Sharing the lessons learned at this point in time may help others to better tailor their own programme to their context.

### Future evaluation

Although the CCFE is considered to be a programme with great potential, more quantitative evidence is needed to secure its sustainability. Therefore, we have designed a prospective quasi-experimental study comparing the programme to usual care on the Triple Aim. This study is a Multi-Criteria Decision Analysis in which patient-reported outcomes are not only measured, but also weighted by their importance to different stakeholders [41].

## Conclusion

This study presents essential success factors of implementing an integrated care programme for community-dwelling frail elderly, the CCFE. These success factors include the holistic assessment of unmet health and social care needs, direct engagement of the patient in the multidisciplinary team meetings, strong leadership by the care groups, close collaboration with the healthcare insurer, a bundled payment, a shared ICT-system and a shared desire to continuously improve. The extent to which these factors are transferable to other settings depends on the context. However, our general recommendations for implementing a similar intervention in different health and social care systems are to adopt an incremental growth approach, involve a GP-role that is responsible for building a team culture and maintaining close relationships with both the patient and the social care sector, establish an integrated way of financing that secures budgets for a longer term, and design a shared information system to accommodate a smooth collaboration between all professionals involved in the programme.

## Additional Files

The additional files for this article can be found as follows:

10.5334/ijic.4626.s1 Appendix A.1 Interview partner overview.

10.5334/ijic.4626.s2 Appendix A.2 Thematic focus areas for interviews.

10.5334/ijic.4626.s3 Appendix A.3 The SELFIE Framework for Integrated Care for Multi-Morbidity.
