# Supplementary material for: Integrated Care for Frail Elderly: A Qualitative Study of a Promising Approach in The Netherlands
Source: Int J Integr Care. 2019 Sep 3;19(3):16. doi: 10.5334/ijic.4626 (PMC6729107; doi:10.5334/ijic.4626)
Supplement: Appendix A.1 — Interview partner overview. [file ijic-19-3-4626-s1.pdf]

(6)Appendix

A.1: Interview partner overview

**Reference in text**	**Stakeholder description**
**PM_1**	**P**rogramme **m**anager/ Initiator
**PM_2**	**P**rogramme **m**anager
**PM_3**	**P**rogramme **m**anager
**IN_1**	**In**itiator
**IN_2**	**In**itiator/ Other stakeholder
**HI_1**	Representative of the sponsor/payer (**H**ealth **i**nsurer)
**HI_2**	Representative of the sponsor/payer (**H**ealth **i**nsurer)
**MS_1**	Non-physician **m**edical **s**taff
**MS_2**	Non-physician **m**edical **s**taff
**IC_1**	**I**nformal **c**aregiver
**FE_1**	Patient (**F**rail **E**lderly)
Refused participation due to time constraints	Physician
Refused participation due to time constraints	Representative of the sponsor/payer (Health insurer)
