# Supplementary material for: Integrated Care for Frail Elderly: A Qualitative Study of a Promising Approach in The Netherlands
Source: Int J Integr Care. 2019 Sep 3;19(3):16. doi: 10.5334/ijic.4626 (PMC6729107; doi:10.5334/ijic.4626)
Supplement: Appendix A.2 — Thematic focus areas for interviews. [file ijic-19-3-4626-s2.pdf]

**(6)Appendix**

**A.2:** Thematic focus areas for interviews

| Stakeholder \ Task | | T2 | T3 | T4 | T5 | T6 | T7 | T8* | TS** |
|---|---|---|---|---|---|---|---|---|---|
| | | Implementation process and barriers to implementation | Design of delivery of care including relationships between partners involved | Partnerships beyond the health care system | Use of ICT applications | Use of self-management interventions | Involvement of new professional roles | Evaluation | Financing and payment schemes |
| A. | Manager(s) | X | XX | XX | X | XX | XX | X | X |
| B. | Initiator(s) | XX | XX | X | o | X | XX | X | X |
| C. | Representative(s) of sponsor/payer organisations | XX | o | X | o | o | o | X | XX |
| D1. | Physician(s) | o | XX | XX | X | X | X | X | X |
| D2. | Non-physician medical staff (e.g. nurses), social staff, new professional groups (if applicable) | o | XX | XX | XX | XX | XX | X | X |
| E. | Informal caregiver(s) | o | XX | XX | XX | XX | o | o | o |
| F. | Client(s) or their representative(s) | o | XX | XX | XX | XX | XX | o | o |
| G. | (Other stakeholder(s)) | *Interview protocol to be developed only if necessary and based on protocols of the stakeholder types closest to this stakeholder* | | | | | | | |

**Note:** XX = main question; X = to be asked only if not mentioned in the answers to main questions; o = not necessary to be asked

* results of T8 mostly derived from document analysis; in the interviews, we want to know about the experience with evaluation/monitoring

** results of TS partly derived from document and literature analysis
