# Supplementary figures and images for: Integrated Care for Frail Elderly: A Qualitative Study of a Promising Approach in The Netherlands
Source: Int J Integr Care. 2019 Sep 3;19(3):16. doi: 10.5334/ijic.4626 (PMC6729107; doi:10.5334/ijic.4626)

## (6)Appendix

### A.3: The SELFIE Framework for Integrated Care for Multi-Morbidity

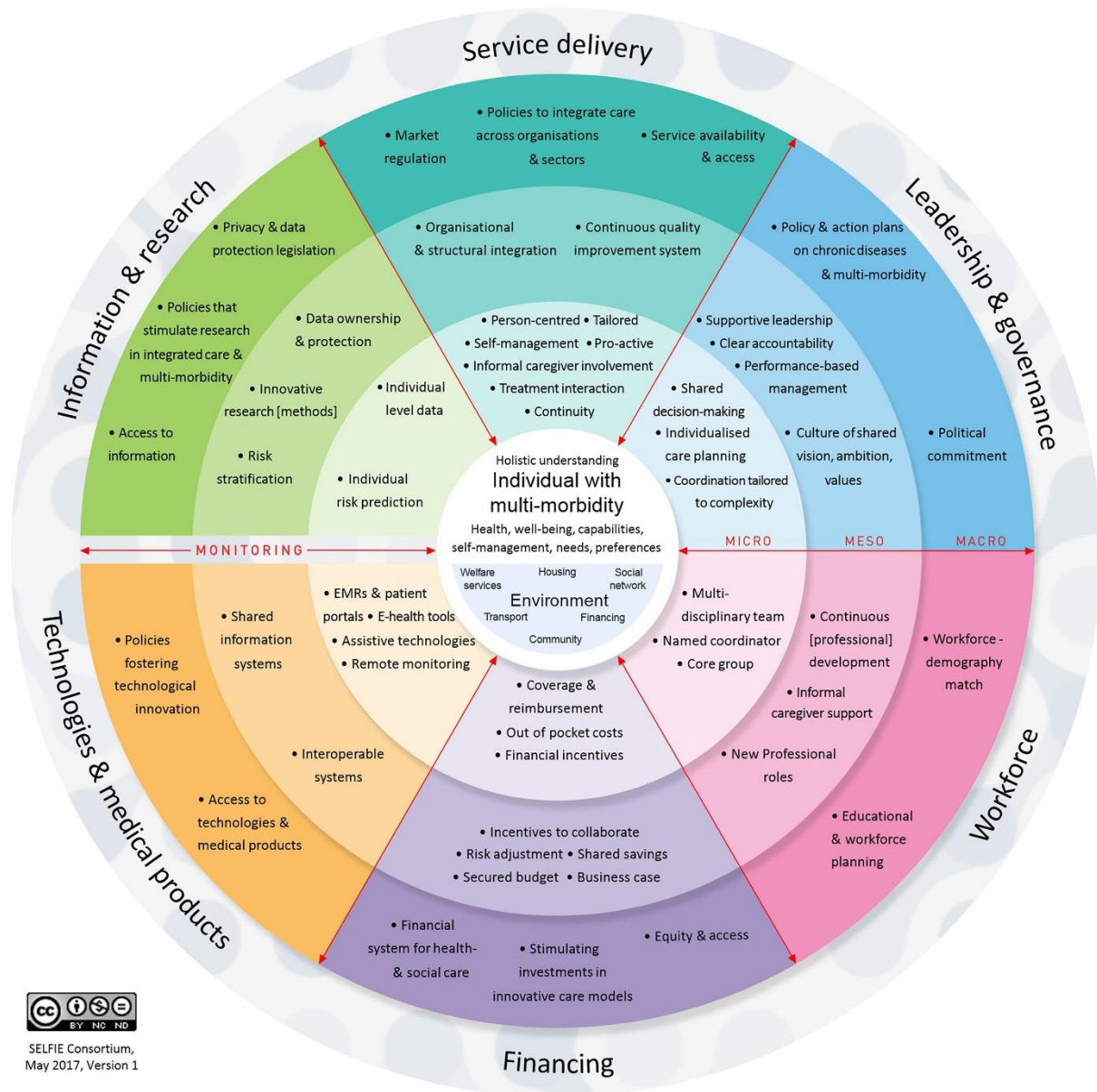

Supplement: Appendix A.3 — The SELFIE Framework for Integrated Care for Multi-Morbidity. [file ijic-19-3-4626-s3.pdf]
